# cGMP Signaling in the Cardiovascular System—The Role of Compartmentation and Its Live Cell Imaging

**DOI:** 10.3390/ijms19030801

**Published:** 2018-03-10

**Authors:** Nadja I. Bork, Viacheslav O. Nikolaev

**Affiliations:** 1 Institute of Experimental Cardiovascular Research, University Medical Center Hamburg-Eppendorf, University of Hamburg, Hamburg 20246, Germany; n.bork@uke.de; 2 German Center for Cardiovascular Research (DZHK), Partner site Hamburg/Kiel/Lübeck, Hamburg 20246, Germany

**Keywords:** cGMP, cardiovascular system, imaging, FRET, compartmentation

## Abstract

The ubiquitous second messenger 3′,5′-cyclic guanosine monophosphate (cGMP) regulates multiple physiologic processes in the cardiovascular system. Its intracellular effects are mediated by stringently controlled subcellular microdomains. In this review, we will illustrate the current techniques available for real-time cGMP measurements with a specific focus on live cell imaging methods. We will also discuss currently accepted and emerging mechanisms of cGMP compartmentation in the cardiovascular system.

## 1. cGMP Signaling in the Cardiovascular System

The ubiquitous second messenger cyclic guanosine 3′,5′-monophosphate (cGMP) plays an important role in the cardiovascular system [1,2,3,4]. Since its discovery more than 50 years ago [5], a lot of research has been done on cGMP signaling which still represents an actively studied topic [6]. In the cardiovascular system, cGMP signaling is essential to several cell types, including cardiomyocytes (CMs), vascular smooth muscle cells (VSMCs), endothelial cells (ECs), and cardiac fibroblasts (CFs) [1,7,8,9,10].

### 1.1. cGMP Synthesis

cGMP is generated from guanosine triphosphate (GTP) by specialized enzymes called guanylyl cyclases (GCs). There are at least two different pathways for cGMP formation—one stimulated by nitric oxide (NO) and another triggered by natriuretic peptides (NPs) [11,12].

In mammalian cells and tissues, GCs exist in two different forms. The soluble guanylyl cyclase (sGC) is a haem-containing enzyme, consisting of two subunits (α and β, the latter contains a haem binding domain). The particulate guanylyl cyclase (pGC) is a single-chain haem-free transmembrane protein. Both GCs are involved in cGMP signaling [12,13,14,15]. sGCs are stimulated by NO [16,17,18], whereas pGCs are stimulated by NPs [19,20].

There are at least seven pGCs (GC-A to GC-G). However, thus far, ligands for only three of them (GC-A, GC-B, and GC-C) have been identified, whereas the other pGCs are supposed to act as orphan receptors [19,21]. Atrial natriuretic peptide (ANP) and brain natriuretic peptide (BNP) are the natural ligands for GC-A. C-type natriuretic peptide (CNP) is the ligand for GC-B [9,21,22,23,24]. pGCs are localized to cell membranes [25].

sGC consists of two subunits and is typically found as a heterodimer composed of a larger α subunit and a smaller β subunit. sGCα_1_β_1_ heterodimer is the most prevalent sGC isoform, although homodimers of these subunits can also be formed [1,13,15,26]. Indeed, sGC has also been shown to be targeted to caveolin-rich membrane domains together with NO-producing synthases (NOS). For example, the co-localization with caveolin-1 [27], heat shock protein 90, and endothelial NO synthase (eNOS) was demonstrated in aortic ECs [28], as well as the membrane association of sGCα_2_β_1_ in rat brain [14].

cGMP concentrations can be also modulated by stimulation with acetylcholine (ACh). There are two major types of ACh receptors (AChRs), the muscarinic AChRs (mAChRs) and the nicotinic AChRs (nAChRs). [29,30,31]. ECs, smooth muscle cells, as well as CMs are known to express both, mAChRs and nAChRs [32,33,34,35,36,37]. Studies in the early 1990s could already show that stimulation of blood vessels with ACh leads to an endothelium-dependent relaxation of the vascular smooth muscle which is mediated through the formation of an endothelium derived relaxing factor (EDRF) which has been identified as NO [38,39]. EDRF can be produced by NOS enzymes in ECs upon ACh stimulation of the muscarinic M_3_ receptors, this pathway leads to an increase cGMP levels of vascular smooth muscle by stimulation of sGC [40,41,42]. In the heart, muscarinic M_2_ receptors expressed in atrial CMs play a crucial role in the regulation of heart rate and arhythmogenesis [43], which is mediated by ventricular CMs, since they are partially innervated by the parasympathetic nervous system [44]. Interestingly, nAChRs are also expressed in myocardium and can mediate protection of the heart from ischemia reperfusion injury [45].

### 1.2. cGMP Effector Activation

There are at least three classes of presently known cGMP effector proteins. First, cGMP-dependent protein kinases (PKGs, also known as cGKs), are important downstream targets of cGMP in the cardiovascular system. Three PKG isoforms have been identified (PKG-type Iα (PKG Iα), PKG-type Iβ (PKG Iβ), and PKG-type II (PKG II)), whereby PKG Iα and PKG Iβ are splice variants originating from alternative splicing of one single gene. In the cardiovascular system, PKG I is the major isoform—PKG Iα and PKG Iβ are expressed in VSMCs—while ECs express PKG Iβ, and CMs express PKG Iα [1,26,46,47,48,49,50]. Second, cyclic nucleotide-gated (CNG) channels, nonselective cation channels, can be activated by the binding of cGMP or cyclic adenosine 3′,5′-monophosphate (cAMP). They play a central role in the signal transduction pathways of vision and olfaction, as well as in the regulation of sinus node function and cardiac pacemaking [51,52,53]. Finally, activity of some phosphodiesterases (PDEs) can be regulated by cGMP as described below.

### 1.3. cGMP Catabolism by Phosphodiesterases

Cyclic nucleotide PDEs hydrolyze cyclic nucleotides, e.g., cGMP and cAMP to inactive monophosphates guanosine monophosphate (GMP) and adenosine monophosphate (AMP), respectively. Therefore, they are involved in the regulation of the cellular levels of the second messengers cAMP and cGMP [13,25,50,54,55,56].

At least 21 genes encoding for PDEs have been described. PDEs can be subdivided into 11 families (PDE1–PDE11) [57,58]. In the heart, seven PDE families have been investigated (PDE1, PDE2, PDE3, PDE4, PDE5, PDE8, and PDE9). While PDE1, PDE2, PDE3, PDE10, and PDE11 are dual-substrate specific and can hydrolyze both cAMP and cGMP, PDE4, PDE7 and PDE8 are specific for cAMP, whereas PDE5, PDE6 and PDE9 can only hydrolyze cGMP. Dual-substrate specific PDEs, especially PDE2 and PDE3 lead to a crosstalk between cAMP and cGMP. PDE3 primarily hydrolyzes cAMP (V_max_ for cAMP 4–10 times higher than for cGMP [59]), but can be competitively inhibited by cGMP. Therefore, it is often referred to as the cGMP-inhibited PDE. In that way, increasing cGMP concentrations (which inhibit PDE3) can increase cAMP levels leading to the so-called positive cGMP-to-cAMP cross-talk. PDE2 can be allosterically activated by cGMP and therefore, an increase in cGMP level can negatively regulate cAMP levels via PDE2, creating the so-called negative cGMP-to-cAMP cross-talk [54,59,60,61,62].

## 2. Live Cell Imaging of cGMP

Since a lot of research is done on cGMP signaling, the development of techniques to measure cGMP concentrations in living cells and tissues has become essential. Up to now, several techniques to measure cGMP concentrations have been developed and applied in numerous studies [6,63,64] (Table 1).

Traditional biochemical methods such as immunohistochemistry [65], radioimmunoassays, enzyme-linked immunoassays (ELISA) [66,67] or Western blot analysis [64] are quite sensitive and specific methods to detect cGMP or its downstream effector function. However, they all represent cell-destructive type of assays which can measure only total cGMP levels with low spatial resolution instead of physiologically relevant free cGMP localized in subcellular microdomains. In addition, they often require PDE inhibition to obtain adequate sensitivity, thereby ignoring tight regulation of local cGMP by these enzymes [64,68]. In sharp contrast, development of real-time detection methods during the last two decades has enabled studies of cyclic nucleotide dynamics and compartmentation in living cells with high temporal and spatial resolution in real time [64] (Table 1).

The first real-time detection of cGMP in living cells was done by electrophysiological recordings of CNG ion channels. For their experiments, Trivedi and Kramer [69] used an exogenously expressed CNG channel that was engineered to be especially sensitive and selective for cGMP in neoblastoma cells [69,70]. CNG measurements were also used in adult rat CMs [71]. For this purpose, the rat olfactory CNG channel α-subunit was expressed in adult rat CMs, and recordings of the respective cGMP-gated current (I_CNG_) was done [69,71]. The use of CNG channels for live cell measurements offers the advantage of high temporal resolution. However, electrophysiological measurements are technically challenging and time consuming and their temporal resolution is rather limited to subsarcolemmal microdomains [72].

Förster resonance energy transfer (FRET) microscopy has become a useful tool to monitor and quantify real-time dynamics of protein–protein interactions and biochemical processes [73]. FRET is the radiationless transfer of excited-state energy from an initially excited fluorescent donor to an acceptor fluorophore molecule [74,75,76]. Up to now, several FRET sensors to measure cGMP dynamics have been described and used in multiple cell types (Table 1).

The sensor CGY-Del1 was one of the first cGMP sensors developed for FRET imaging. It consists of tandem fusion proteins of enhanced cyan (ECFP), PKG I α_Δ1–47_, and enhanced yellow fluorescent protein (EYFP). However, despite very good sensitivity (cGMP EC_50_ value of 20 nM), it has a low cGMP/cAMP selectivity and is not usable in adult CMs which have high cAMP levels [77,78]. Another cGMP FRET sensor developed at the same time was the Cynget-1/2 sensor. It consists of a PKG (with N-terminally deleted residues 1–77), flanked between cyan and yellow fluorescent proteins. With a much better cGMP/cAMP selectivity than the CGY-Del1 sensor, this sensor has been used in neonatal CMs, however it is not usable in adult CMs due to a very low sensitivity (EC_50_ = 1.5/1.9 µM) [68,79]. The use of these sensors (CGY and Cygnets) is limited by either low specificity or sensitivity and low temporal resolution [77].

Two other types of cGMP FRET sensors with faster kinetics and moderate cGMP/cAMP selectivity have been developed based on cGMP binding regulatory domains of PDEs or cGMP binding domains of PKG. One is the cGES-DE2/5 FRET sensor. It consists of a PDE2A GAF-B domain or PDE5A GAF-A domain fused to ECFP and EYFP [77,80]. Another group of constructs are the cGi-FRET sensors (cGi-500/3000/6000). In these sensors, both cGMP-binding domains from PKG I are sandwiched between ECFP and EYFP [81,82,83]. However, with cGMP EC_50_ = 0.9/1.5 µM for cGES-DE2/5, and cGMP EC_50_ = 500/3000/6000 nM for cGi-500/3000/6000 (cAMP EC_50_ values are in the range of 100–500 µM), neither is optimal for adult CMs because of relatively low sensitivity.

Because of very low cGMP concentrations in adult CMs, reliable cGMP measurements in these cells have been challenging in the past [63]. With the development of the red cGES-DE5 FRET sensor, consisting of a single cGMP-binding (GAF-A) domain from PDE5 fused to the fluorophores T-Sapphire and Dimer2 and an EC_50_ of ~40 nM, cGMP FRET measurements in adult CMs became possible [63,84,85].

It is important to mention that there is also a non-FRET type of cGMP sensors called FlincG1-3, which can be used for cGMP measurements as well. FlincGs are composed of both cGMP binding domains from PKG I fused to a circularly permuted GFP. However, despite a good sensitivity for cGMP with EC_50_ = 0.17–0.89 µM, they have a relatively low cGMP/cAMP selectivity [86,87] (Table 1).

## 3. Compartmentation of cGMP Signaling in the Cardiovascular System

Caused by the lack of real-time cGMP detection methods in the past, spatial localization of the cGMP signaling pathway components has long been poorly studied [6]. However, with the development of methods for real-time cGMP detection, it became possible to analyze cGMP compartmentation in intact cells of the cardiovascular system.

### 3.1. Compartmentation in Cardiomyocytes

In CMs, cGMP signaling is involved in the regulation of contractility [1]. Various studies have been done, investigating the effects of NP/pGC/cGMP- and NO/sGC/cGMP-pathways on CM contractility.

CNP was shown to have a direct positive chronotropic [88] and dromotropic [89] effect in anaesthetized dogs. Studies in cultured neonatal rat CMs showed negative inotropic effects caused by CNP stimulation [90,91]. In rat papillary muscles, CNP exerted a positive lusitropic and a negative inotropic effect [92]. In isolated working mouse hearts, CNP exerted positive inotropic and lusitropic effects [93,94], followed by a delayed negative inotropic action [93].

ANP effects on CM contractility seem to be less clear. Some studies could show that ANP decreases contractility in isolated CMs [95,96], whereas in other studies no effect of ANP on cardiac mechanical function could be detected [89,92]. One study showed that ANP has no direct effects on cardiac contractility in isolated working mouse hearts, but the chronic absence of its receptor, GC-A, results in increased responsiveness to CNP [93].

The effect of NO on the heart has also been investigated by various groups. While some studies found a negative inotropic effect of NO donors on force contraction in human atrial and ventricular myocardium [97] and in isolated adult rat ventricular CMs [98], other studies could not observe any effects of NO donors in isolated cat and rat papillary muscles [99], or in atrial myocardium preparations from rats, rabbits, guinea pigs, frogs, and humans [100].

One possible explanation for the different effects of NO donors in different studies was given by Wegener et al. [101]. They showed that, in atrial and ventricular muscle strips, myoglobin acts as intracellular scavenger of NO, preventing NO from reaching its intracellular receptors in CMs. Therefore, the different NO effects seem to be—at least in part—dependent on the myoglobin concentration in the particular preparations [101].

**Figure 1:**
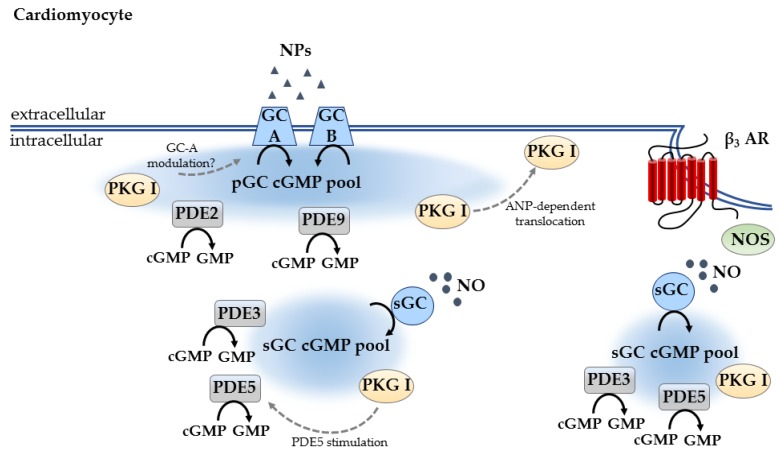
cGMP compartmentation in CMs. There are at least two different cGMP pools: the NP/pGC/cGMP pool formed at the plasma membrane, and the NO/sGC/cGMP pool which can be controlled by the β_3_-adrenergic receptor (β_3_-AR) at the cell surface caveolae. pGC/cGMP is tightly controlled by PDE2 and PDE9, while sGC/cGMP pool is predominantly regulated by PDE5 and PDE3. PKG I is one of the major downstream targets for cGMP signaling. NO/sGC stimulated PKG I is known to phosphorylate and inhibit PDE5. ANP/pGC stimulated PKG I can be recruited to the plasma membrane and is assumed to modulate GC-A activity. Black arrows indicate enzymatic activity, dotted arrows indicate protein action.

In 2006, a cell-based study provided the first evidence that the cGMP signaling pathways are compartmentalized [71]. It was already known before from a previous report which used frog ventricular myocytes to study the modulation of I_Ca_ by NO donors, that the NO-mediated signaling remains in the local environment and is closely associated with local cAMP concentrations, implying the formation of signaling microdomains [102]. The group around Rodolphe Fischmeister [71] tested whether the different effects of NPs and NO donors on cardiac and vascular smooth muscle function are due to an intracellular compartmentation of cGMP. In rat CMs, they monitored subsarcolemmal cGMP signals by ectopically expressed rat olfactory CNG channel α subunit and real-time recordings of the associated cGMP-gated current (I_CNG_). They could show that in rat CMs, the particulate (ANP-stimulated) cGMP pool is readily accessible at the plasma membrane, whereas the soluble (NO donor-stimulated) pool is not. Additionally, they showed that PDE5 controls the soluble but not the particulate pool, whereas the latter is under the exclusive control of PDE2 (Figure 1) [71,103]. However, the study did not examine whether this compartmentation resulted in different PKG and/or cellular functional response.

The effect of PKG on the different cGMP pools generated by pGC and sGC was investigated some years later. In adult CMs, it was shown that PKG activation limits the accumulation of cGMP induced by NO donors (via PDE5 stimulation) but increases that induced by NPs (by a still unknown mechanism, possibly by PKG-dependent modulation of GC-A). This indicates that PKG, via a feedback control, is one of the key components in the modulation of subcellular microdomains [104].

There are also some hints that PKG is recruited to the plasma membrane, even though such studies have not been done in CMs yet. It could be shown in a yeast two-hybrid system, that PKG can directly interact with GC-A. In human embryonic kidney cells expressing GC-A, it is recruited to the plasma membrane following ANP treatment. In this system, PKG translocation was ANP-dependent but not NO dependent [105]. The same effect could also be shown in rat hepatocytes where ANP promotes plasma membrane recruitment of PKG Iα through localized cGMP elevation [106].

Another study examined the functional significance of such cellular compartments which were found by Liliana Castro and colleagues [71]. In an in vivo study, Takimoto and colleagues could show that the regulation of cardiac β-adrenergic response by cGMP is linked to a NO-synthesis/PDE5-hydrolyzed pool signaling via PKG. In contrast to that, NP stimulation achieved greater detectable increases in cGMP but not PKG activity and did not modulate β-adrenergic response [107].

It was already known from cardiac function studies in adult mice that PDE5 regulation of the adrenergic response depends upon NOS-induced cGMP/PKG and can be enhanced by sustained low-level stimulation of sGC [108]. To investigate the cellular mechanisms for the modulation of the β-adrenergic response by PDE5 inhibition, another study was done in adult mouse CMs. The authors examined the role of PDE2 and PDE3, β_3_-adrenergic signaling, and PKG targeting of the contractile apparatus for modulation of β-adrenergic response by PDE5 inhibitors. They could show that anti-adrenergic effects of PDE5A inhibition are not modulated by PDE2 or PDE3, but rather require β_3_-AR stimulation and PKG activation with a subsequent Troponin I phosphorylation [109].

With the development of the red cGES-DE5 FRET sensor it became possible to perform FRET measurement in adult mouse CMs. Studies in transgenic mice with CM-specific expression of this cytosolic FRET biosensor showed that PDE3 is the main PDE responsible for cytosolic cGMP degradation [63]. In 2015, a study was presented which investigated the involvement of cGMP degrading PDE9 in the heart [110]. The role of PDE5 as regulator of NO-generated cGMP was already known [103], however PDEs controlling NP-generated cGMP were still uncertain at this time. The authors could show that in adult mouse hearts and in isolated CMs, PDE9 regulates NP- rather than NO-stimulated cGMP [110] (Figure 1).

Despite these several studies investigating cGMP compartmentation in CMs, yet there are still many unresolved questions. For further studies of cGMP microdomains in adult CMs, targeted cGMP FRET sensors-like those which already exist for cAMP [111,112]-would be especially useful.

### 3.2. Compartmentation in Other Cell Types

#### 3.2.1. Compartmentation in Vascular Smooth Muscle Cells

In VSMCs, cGMP signaling is involved in cell proliferation and differentiation [1,113]. VSMCs are the contractile cells of the blood vessels including the coronary arteries and veins [47] and it could be shown that the NO/cGMP signaling cascade plays an essential role in vascular smooth muscle relaxation [114,115].

In 2006, a study could show that several differences might exist between cGMP signaling in CMs and VSMCs. The authors investigated the subcellular localization of cGMP signals by measuring CNG channel activity in response to agonists for either pGC or sGC in human embryonic kidney cells expressing GC-A as well as in VSMCs. They could show that cGMP signals are spatially segregated and that the functional compartmentation of cGMP signals may underlie the unique actions of ANP and NO [116]. Comparing the results from this study with the study from Castro et al. [71], it becomes obvious that, in VSMCs, the relative increase in cGMP with NO-sGC stimulation exceeded that with NP-pGC [116], which is the opposite to what was observed in CMs [71].

**Figure 2:**
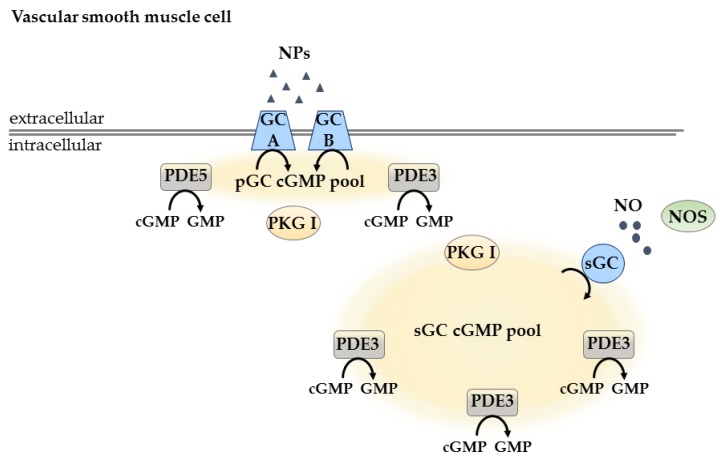
cGMP compartmentation in VSCMs. In VSMCs, there are two different cGMP pools: the pGC/cGMP pool, which is controlled by PDE5 and PDE3, and the sGC/cGMP pool, which is mainly regulated by PDE3. Black arrows indicate enzymatic activity.

In another study, it was shown by the use of the cGMP-biosensor FincGs that, in unpassaged adenoviral transfected VSMCs, global cGMP elevation was created to NO response. In contrast, local sub-membrane elevations were generated in response to ANP, which were converted to global, more diffused ones after PDE5 inhibition [87] (Figure 2).

In human VSMCs transduced with adenoviral vectors to express mutants of rat olfactory CNG channel-subunits, it could be shown by recording cGMP-gated currents (I_CNG_) that there are at least two separate cGMP pools: one localized next to plasma membrane and controlled by PDE5 and PDE3, and another localized to cytosol, regulated mainly by PDE3 [117] (Figure 2).

The development of transgenic cGMP FRET sensor mice, expressing the cytosolic cGi500 cGMP FRET sensor [82] in VSMCs offered new possibilities to study cGMP dynamics in these cells [83]. The authors created two different cGi500 mouse lines-a smooth-muscle specific transgenic line (SM22-cGi500 mice), expressing the cGi500 sensor under the control of the smooth-muscle-specific SM22 α promoter, and the ubiquitous transgenic mouse line (R26-CAG-cGi500(L1) mice), where a targeted knock-in of the cGi500 sensor was done into the Rosa26 locus of Cre recombinase-activatable expression cassette driven by the ubiquitous cytomegalovirus early enhancer/chicken β-actin/β-globin (CAG) promoter with a permanently active sensor transgene. FRET measurements in isolated VSMCs of both mouse lines showed that both CNP superfusion, and superfusion with the NO-releasing compound 2-(*N*,*N*-dethylamino)-diazenolate-2-oxide dethylammonium salt (DEA/NO) generated clearly detectable cGMP increases. Additionally, FRET measurements in VSMCs isolated form SM22-cGi500 mice showed that preincubation with the nonspecific PDE inhibitor 3-isobutyl-1-methylxanthine (IBMX) strongly potentiated NO-induced cGMP signals, whereas the selective PDE5 inhibitor sildenafil hat a comparatively weak effect. This indicates that cGMP levels in VSMCs are controlled by PDE5 and other PDEs. Additionally, differences in response to ANP and CNP were also shown. Whereas CNP stimulation leads to robust increase in cGMP concentrations, stimulation with ANP leads to weak but clearly detectable cGMP elevation [83] (Figure 2).

#### 3.2.2. Compartmentation in Endothelial Cells

While cGMP signaling has been extensively studied in VSCMs and CMs, studies in ECs are only at their beginning. In ECs, cGMP signaling is known to regulate cell motility, migration, and proliferation, which are vital to angiogenesis and vascular permeability [1].

**Figure 3:**
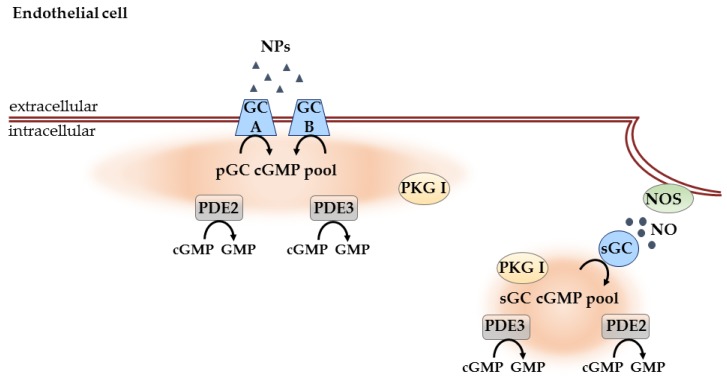
cGMP compartmentation in ECs. These cells also contain pGC and a sGC-associated cGMP pools. Both PDE2 and PDE3 are known to be involved in their regulation. The effect of cGMP signaling pathway is highly dependent on intracellular cGMP concentrations. sGC, PKG and PKA can be also localized to endothelial caveolae to regulate endothelial barrier function. Black arrows indicate enzymatic activity.

The role of cGMP signaling on endothelial permeability has long been controversial. Surapisitchat et al. [118] hypothesized that the effect of cGMP on endothelial permeability is dependent on cGMP concentration. In their studies, the authors could show that in human umbilical vein endothelial cells (HUVECs), after slight elevation of cAMP with MPB-forskolin, low doses of either ANP or NO donors potentiated the inhibitory effect of MPB-forskolin on thrombin-induced permeability caused by inhibition of PDE3A at lower cGMP concentrations. However, this inhibitory effect was reversed at higher doses of ANP and NO donor because cGMP at higher concentrations activates PDE2A. These findings suggest that the result of cGMP signaling in endothelial cell permeability is highly dependent on the concentration of intracellular cGMP [118] (Figure 3).

Some years later, another study was done by Chen et al. [119] which extended the previous findings by Surapisitchat et al. [118] that the effect of the cGMP signaling pathway in ECs is highly dependent on the intracellular cGMP concentrations. By doing real-time FRET measurements in HUVECs, the authors analyzed the effects on the regulation of submembrane versus cytosolic cAMP levels by ANP stimulation, using the membrane-targeted cAMP sensor pmEpac2-camps [112] or the cytosolic cAMP sensor Epac2-camps [120]. Whereas in resting HUVECs, ANP leads to an increase in submembrane and cytosolic cAMP levels indicating inhibition of the cGMP inhibited PDE3A, in HUVECs pretreated with TNF-α which induces PDE2 expression in these cells, ANP treatment mediated a mild but clear decrease of submembrane cAMP level (which was PDE2A mediated) but the effect of ANP on cytosolic cAMP level was unchanged. With these measurements, the authors could show that the cGMP/cAMP cross-talk is compartmentalized in ECs. [119] (Figure 3).

Another study provided further evidence of compartmentation of sGC, PKG and protein kinase A (PKA) in endothelial cell caveolae. It is known that, similar to CMs, in ECs, endothelial NO synthase (eNOS) is localized to caveolae. Using endothelium-intact aortic rings, relaxation of pre-contracted (with ACh) vessels by the sGC activator YC-1 and by 8-bromo-cGMP was impaired in the presence of methyl-β-cyclodextrin—a chemical agent that disassembles caveolae by sequestering cholesterol from the membrane [27]. This suggests that sGC/cGMP/PKG pathway compartmentation in caveolae is required for the functional response of the vessels (Figure 3).

#### 3.2.3. Compartmentation in Cardiac Fibroblasts

CFs are the most abundant non-myocyte cell type in the heart. They help to maintain the extracellular matrix (ECM) of the heart by producing ECM components such as collagen and fibronectin, as well as promoting collagen degradation by secreting matrix metalloproteinases [7,121,122,123]. Even though CFs are the most abundant non-myocyte cell type in the heart, little is known about cGMP compartmentation in these cells.

A quantitative analysis of the cardiac fibroblast transcriptome revealed that CFs express components of the NO/cGMP signaling pathway—the α_1_ and β_1_ sGC subunits, and PKG I. Inhibition of the proliferation of serum-treated CFs upon cGMP analog treatment indicates that PKG I mediates the inhibitory effects of the NO/cGMP pathway on CF growth [124].

One study was done to investigate the effect of NO production on β-adrenergic response in adult rat CFs. Upon immune response activation by IL-1β treatment, the inducible NO synthase (iNOS) is upregulated, which increases NO and therefore cGMP production to attenuate cAMP accumulation in response to isoproterenol or forskolin. By using of the broadband PDE inhibitor IBMX and the PDE2 specific PDE inhibitor erythro-2-(2-hydroxy-3-nonyl)adenine, the authors could show that cAMP attenuation is regulated via PDE2 stimulation caused by increased cGMP levels and not via an inhibition of adenylyl cyclase by NO or via stimulation of PKG [125].

An outside-in-crosstalk between the ECM protein fibronectin and GC-A has been also recently described. In cultured human CF, it could be shown that GC-A might transduce signals from ECM to CF. CFs plated on fibronectin demonstrated an increase in cGMP production to BNP compared to non-coated plates. This indicates that GC-A interacts with ECM components such as fibronectin to enhance BNP activation of cGMP [122]. It could be also shown that both Arg-Gly-Asp (RGD) attachment site containing ECM proteins and integrins may interact with BNP/GC-A to modulate cGMP generation [126].

## 4. Conclusions

During the last decades, a lot of research has been done on cGMP signaling in the cardiovascular system. However, there is still a plethora of unresolved questions. New imaging techniques for real-time cGMP detection offer great possibilities to better understand the role of subcellular compartmentation of cGMP signaling in living cells and tissues and until now, several interesting studies have been done investigating the role of subcellular cGMP compartmentation in the cardiovascular system.

Thus far, only a few studies focusing on subcellular cGMP compartmentation in adult CMs have been performed, mostly limited by the availability of highly sensitive live cells imaging techniques. It is known that in CMs there are at least two different cGMP pools: the NP/pGC/cGMP pool, controlled by PDE2 and PDE9, and the NO/sGC/cGMP pool, predominantly regulated by PDE5 and PDE3. One of the main downstream targets is PKG I, it is known to phosphorylate and inhibit PDE5. ANP/pGC stimulated PKG I can be recruited to the plasma membrane and is assumed to modulate GC-A activity. Several questions remain unresolved such as the role of GC distribution in the regulation of cGMP compartmentation. For such studies, the development of targeted cGMP FRET biosensors (similar to those which already exist for cAMP) would be very useful.

Studies in VSMCs showed the existence of two different cGMP pools: pGC/cGMP, controlled by PDE5 and PDE3, and sGC/cGMP, mainly regulated by PDE3. Thus far, most of the studies have been done using electrophysiological recordings of CNG channel activity, which are limited to the subsarcolemmal microdomains. In ECs, only a few studies have been done to show that the effect of cGMP signaling pathway is highly dependent on the intracellular cGMP concentrations. pGC- and a sGC-associated cGMP pool have been identified and shown to be regulated and PDE2 and PDE3. Recent reports suggest interesting PDE2/PDE3-mediated compartmentation of cGMP at the membrane vs. other subcellular locations which should be further address using FRET biosensors.

Although new imaging techniques for real-time cGMP detection offer great possibilities to better understand the role of cGMP compartmentation, such studies remain challenging. Low cGMP concentration and complexity of subcellular microdomains require special efforts and the development of better techniques and biosensors, which is still ongoing. This is a challenging but important field of research since a better understanding of cGMP compartmentation in the cardiovascular system could offer novel pharmacological approaches for the treatment of multiple cardiovascular diseases.

## Figures and Tables

**Table 1:** Techniques to measure cGMP in living cells and tissues.

Method	Advantages/Disadvantages	References
Traditional biochemical methods		
Immunohistochemistry	Quite sensitive and specific	[64,65,66,67]
Radioimmunoassays	Cell destructive assays	
Enzyme-linked immunoassays	Only measure total cGMP levels	
Immunoblots for PKG substrate phosphorylation	Often require PDE inhibition to obtain adequate sensitivity	
Real-time cGMP detection		
Electrophysiological recordings of CNG ion channels	High temporal resolution Technically challenging and time consuming Temporal resolution limited to subsarcolemmal microdomains	[69,70,71]
Förster resonance energy transfer (FRET) based cGMP sensors CGY-Del1 Cynget-1/2 cGES-DE2/5 cGi-500/3000/6000 red cGES-DE5	High temporal and spatial resolution Sensitivity for cGMP measurements in some cell types challenging cGMP/cAMP selectivity important	[77,78] [68,79] [77,80] [81,82,83] [63,84,85]
Non-FRET based cGMP sensors	Good cGMP sensitivity	[86,87]
FlincG1-3	Relatively low cGMP/cAMP selectivity	

## Abbreviations

ACh	Acetylcholine
AChR	Acetylcholine receptor
AMP	Adenosine monophosphate
ANP	Atrial natriuretic peptide
BNP	Brain natriuretic peptide
CAG	Cytomegalovirus early enhancer/chicken β-actin/β-globin
cAMP	Adenosine monophosphate
CF	Cardiac fibroblast
cGMP	Cyclic guanosine 3′,5′-monophosphate
CM	Cardiomyocyte
CNG	Cyclic nucleotide gated channels
CNP	C-type natriuretic peptide
DEA/NO	2-(*N*,*N*-dethylamino)-diazenolate-2-oxide dethylammonium salt
EC	Endothelial cell
ECFP	Enhanced cyan fluorescent protein
ECM	Extracellular matrix
EDRF	Endothelium derived relaxation factor
ELISA	Enzyme-linked immunoassay
eNOS	Endothelial NO synthase
EYFP	Enhanced yellow fluorescent protein
FRET	Förster resonance energy transfer
GC	Guanylyl cyclase
GMP	Guanosine monophosphate
GTP	Guanosine triphosphate
HUVEC	Human umbilical vein endothelial cell
IBMX	3-isobutyl-1-methylxanthine
mAChR	Muscarinic acetylcholine receptor
iNOS	Inducible NO synthase
nAChR	Nicotinic acetylcholine receptor
NO	Nitric oxide
NOS	NO synthase
NP	Natriuretic peptide
PDE	Phosphodiesterase
pGC	Particulate guanylyl cyclase
PKG	cGMP-dependent protein kinase
PKG I	cGMP-dependent protein kinase type I
PKG II	cGMP-dependent protein kinase type II
RGD	Arg-Gly-Asp
sGC	Soluble guanylyl cyclase
VSMC	Vascular smooth muscle cell
